# Draft Genome Sequence of an Attenuated *Streptococcus agalactiae* Strain Isolated from the Gut of a Nile Tilapia (*Oreochromis niloticus*)

**DOI:** 10.1128/genomeA.01627-16

**Published:** 2017-02-09

**Authors:** Ze Zhang, Angen Yu, Jiangfeng Lan, Yulei Zhang, Hua Zhang, Yuhui Li, Minqiang Hu, Jiewei Cheng, Shun Wei, Li Lin

**Affiliations:** a Department of Aquatic Animal Medicine, Research Center for Marine Biology, College of Fisheries, Huazhong Agricultural University, Wuhan, China; b School of Life Sciences, Beijing Normal University, Beijing, China; c National Institute of Biological Sciences, Beijing, China; d State Key Laboratory of Marine Resource Utilization in South China Sea, Key Laboratory of Tropical Biological Resources of Ministry of Education, Hainan Key Laboratory for Sustainable Utilization of Tropical Bioresources, College of Marine Science, Hainan University, Haikou, China

## Abstract

*Streptococcus agalactiae* is a pathogen that causes severe anthropozoonosis within a broad range of hosts from aquatic animals to mammals, including human beings. Here, we describe the draft genome of *S. agalactiae* HZAUSC001, a low-virulent strain isolated from the gut of a moribund tilapia (*Oreochromis niloticus*) in China.

## GENOME ANNOUNCEMENT

*Streptococcus agalactiae*, also known as group B streptococcus (GBS), is a pathogen causing severe anthropozoonosis. In aquaculture sectors, especially tilapia culturing, *S. agalactiae* has caused enormous economic losses and contributes to high mortality rates (1). Bacterial virulence is related to factors such as the presence of the C protein alpha-antigen gene (*bca*), the choline-binding protein A gene (*cbpA*), and the enterococcal surface protein gene (*esp*) reported in *S. agalactiae* (2). *S. agalactiae* HZAUSC001, a strain isolated from a moribund tilapia, showed attenuated virulence in tilapia. Compared to the highly virulent strain *S. agalactiae* GD201008-001 (3), *S. agalactiae* HZAUSC001 possessed lower virulence with a 50% lethal dose (LD_50_) of 5.07 × 10^8^ CFU/ml. Rapid development of next-generation sequencing technology, coupled with fast improvement of computing power, enables researchers to map a bacterial genome within a short period of time. Here, we sequenced and analyzed the HZAUSC001 strain and did a comparative genomic analysis with velogenic strains.

*S. agalactiae* HZAUSC001 was routinely maintained on brain heart infusion medium at 28°C. Genomic DNA was isolated using the TIANamp bacteria DNA kit (Tiangen, China) and stored at −20°C until use. DNA libraries were constructed using the Nextera DNA sample preparation kit (Illumina, USA). Multiplex libraries of genomic DNA were sequenced (4, 5) on Illumina HiSeq 2500 instruments operated according to the manufacturer’s instructions with about 250-fold coverage. Gene annotation was performed using RAST (Rapid Annotations using Subsystems Technology) (http://rast.nmpdr.org) and further annotated by BLAST against the NCBI nt/nr database with an e-value of 10^−5^. The annotated genome was submitted to GenBank.

In total, 3,429,503 paired-end reads were generated from sequencing. The 80 discontinuous contigs with a length of at least 500 bp were then assembled using SOAPdenovo2, which revealed one chromosome containing 2,024,692 bp with a G+C content of 35.37% and 1,972 coding DNA sequences. The *N*_50_, *N*_90_, and maximum contig length of this assembled genome were 91,340 bp, 25,959 bp, and 287,976 bp, respectively. Similar to most other bacterial genomes, most of the genes (290 in 1,971) were responsible for carbohydrate metabolism, a basic need to sustain the life cycle of the bacterial cell. In addition to these necessary genes, 59 genes were identified to be responsible for virulence, disease, and defense, suggesting that *S. agalactiae* HZAUSC001 was a pathogenic strain. Compared to the genome sequence of the highly virulent strain *S. agalactiae* GD201008-001, three main virulence genes, *bca* (6, 7), *cbpA* (8–10), and *esp* (11–13), were absent in the virulence-attenuated strain *S. agalactiae* HZAUSC001. We assumed that the absence of these three virulent genes might be related to its attenuated traits. Availability of the whole-genome sequence of* S. agalactiae* HZAUSC001 will pave the way for more technical studies of the attenuated properties of the genome.

### Accession number(s).

The whole-genome shotgun project for *S. agalactiae* HZAUSC001 was deposited in GenBank under the accession number LHQU00000000.
